# Supplementary material for: Insights into rumen microbial biosynthetic gene cluster diversity through genome-resolved metagenomics
Source: Commun Biol. 2021 Jun 29;4:818. doi: 10.1038/s42003-021-02331-7 (PMC8241843; doi:10.1038/s42003-021-02331-7)
Supplement: Supplementary file 2 — Supplementary Information [file 42003_2021_2331_MOESM2_ESM.pdf]

SUPPLEMENTARY INFORMATION

Insights into rumen microbial biosynthetic gene cluster diversity through genome-resolved metagenomics

Christopher L. Anderson[1] and Samodha C. Fernando*[1]

[1]Department of Animal Science, University of Nebraska, Lincoln, NE 68583
*Corresponding author: Samodha C. Fernando (samodha@unl.edu)

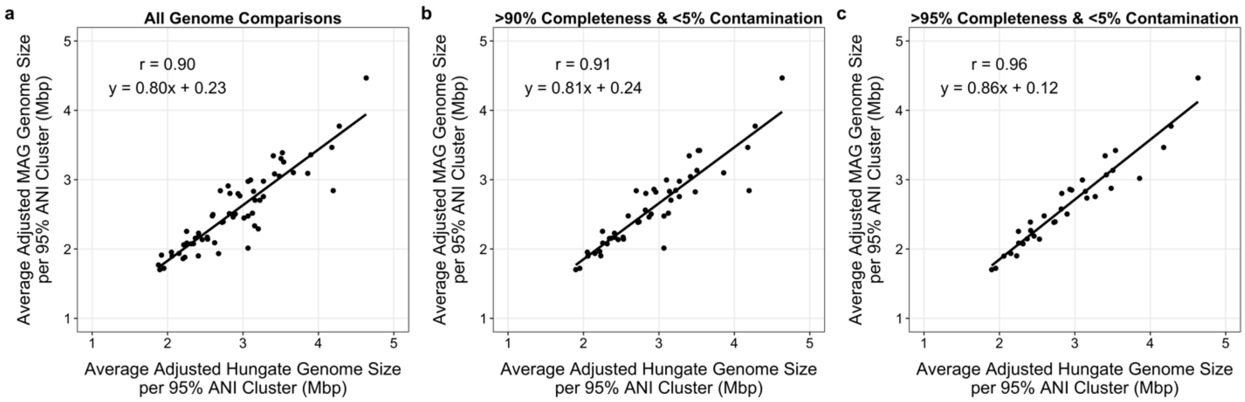

Supplementary Figure 1: The genome sizes of MAGs are largely congruent with genome sizes of isolates from the Hungate1000 Collection in the same 95% ANI cluster. Pearson correlation and linear regression analysis were calculated to compare the average adjusted genome size for all MAGs and Hungate1000 genomes[1] of the same 95% ANI cluster **(a)**, those genomes with ≥90% completeness and ≤5% contamination from the same 95% ANI cluster **(b)**, and those genomes with ≥95% completeness and ≤5% contamination from the same 95% ANI cluster **(c)**.

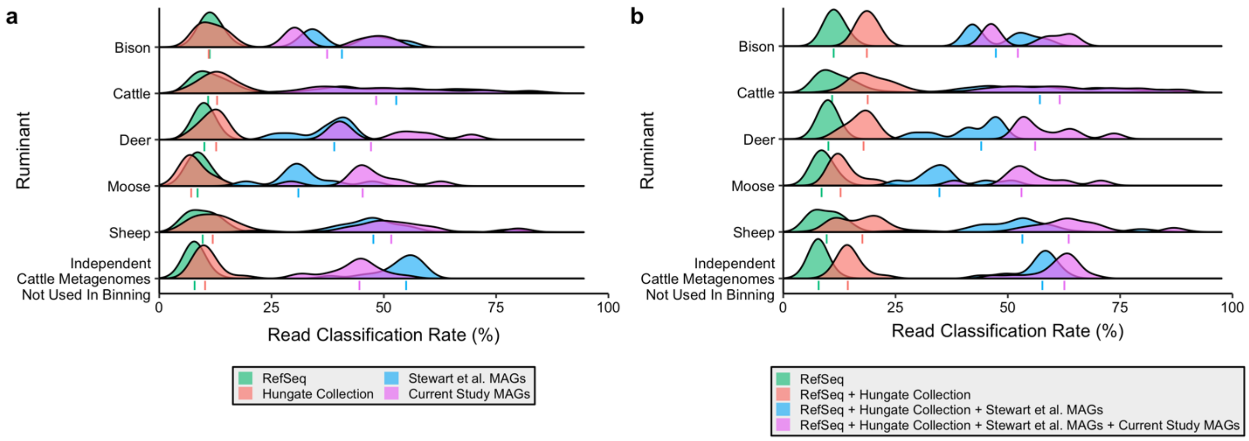

Supplementary Figure 2: Rumen MAGs improve metagenomic classification. Classification rates of reads from the 412 metagenomes used to bin MAGs and 16 cattle metagenomes not applied in genome binning using a combination of genomes from RefSeq, the Hungate1000 Collection[1], Stewart *et al.* studies[2,3], and the current study as databases. The four genomic databases were utilized to classify reads independently **(a)** or used to incrementally build larger databases for classification **(b)**. A database including rumen MAGs from the Stewart *et al.* studies and the current study improved classifications rates for bison, cattle, deer, moose, sheep, and independent cattle metagenomes a median 33.3%, 42.1%, 40.9%, 40.1%, 45.0%, and 46.8% compared to a database of mainly isolate genomes from RefSeq and the Hungate1000 collection. The lines denote the median proportions of sample reads classified by the dataset or combinations of datasets.

Supplementary Table 1: Characteristics and sources of rumen metagenomic datasets used for the reconstruction of MAGs.

Ruminant	QC Bases (Gbp)	Samples	Database	Accession	Publication
Bison	52.3	8	NCBI BioProject	PRJNA214227	NA
Cattle	22.8	16	NCBI BioProject	PRJNA627251	Current Study
Cattle	43.5	27	NCBI BioProject	PRJNA627299	Current Study
Cattle	33.3	23	NCBI BioProject	PRJNA627299	Current Study
Cattle	166.0	1	NCBI BioProject	PRJNA60251	[4]
Cattle	85.1	8	NCBI BioProject	PRJEB10338	[5]
Cattle	52.7	64	MG-RAST	mgp4126	[6]
Cattle	12.5	14	NCBI BioProject	PRJEB8939	[7]
Cattle	60.6	9	NCBI BioProject	PRJNA322715	[8]
Cattle	4.8	1	NCBI BioProject	PRJNA270714	[9]
Cattle	20.9	2	NCBI BioProject	PRJNA291523	[10]
Cattle	125.0	16	NCBI BioProject	PRJNA214227	NA
Cattle	666.0	42	NCBI BioProject	PRJEB21624	[2]
Cattle	73.0	7	NCBI BioProject	PRJNA319009	[11]
Cattle	762.7	82	NCBI BioProject	PRJEB23561	[12]
Deer (White-tailed)	34.2	4	NCBI BioProject	PRJNA214227	NA
Deer (Red)	28.6	4	NCBI BioProject	PRJNA214227	NA
Moose	40.9	3	NCBI BioProject	PRJNA301235	[13]
Moose	67.9	6	NCBI BioProject	PRJEB12797	[14]
Sheep	113.5	16	NCBI BioProject	PRJNA214227	[15]
Sheep	118.3	39	MG-RAST	mgp7948, mgp7949, mgp7950, mgp7957, mgp7958, mgp7959, mgp7960, mgp7961, mgp7962, mgp7963, mgp7964, mgp7965, mgp7966, mgp7967, mgp7968, mgp7969, mgp7970, mgp7974, mgp7975, mgp8090, mgp8091, mgp8092, mgp8093, mgp8094, mgp8095, mgp8096, mgp8097, mgp8098, mgp8099, mgp8108, mgp8109, mgp8110, mgp8111, mgp8112, mgp8113, mgp8114, mgp8115, mgp8116, mgp8117	[16]
Sheep	656.5	20	NCBI BioProject	PRJNA202380	[17]

Supplementary References

1. Seshadri, R. *et al.* Cultivation and sequencing of rumen microbiome members from the Hungate1000 Collection. *Nature Biotechnology* **36**, 359–367 (2018).
2. Stewart, R. D. *et al.* Assembly of 913 microbial genomes from metagenomic sequencing of the cow rumen. *Nature Communications* **9**, 870 (2018).
3. Stewart, R. D. *et al.* Compendium of 4,941 rumen metagenome-assembled genomes for rumen microbiome biology and enzyme discovery. *Nature Biotechnology* **37**, 953–961 (2019).
4. Hess, M. *et al.* Metagenomic Discovery of Biomass-Degrading Genes and Genomes from Cow Rumen. *Science* **331**, 463–467 (2011).
5. Wallace, R. J. *et al.* The rumen microbial metagenome associated with high methane production in cattle. *BMC Genomics* **16**, 839 (2015).
6. Ross, E. M. *et al.* High throughput whole rumen metagenome profiling using untargeted massively parallel sequencing. *BMC Genetics* **13**, 53 (2012).
7. Rubino, F. *et al.* Divergent functional isoforms drive niche specialisation for nutrient acquisition and use in rumen microbiome. *The ISME Journal* **11**, 932–944 (2017).
8. Li, W. *et al.* Metagenomic analysis reveals the influences of milk containing antibiotics on the rumen microbes of calves. *Arch Microbiol* **199**, 433–443 (2017).
9. Kishi, L. T., Jesus, R. B. de, Pavani, C. D., Lemos, E. G. M. & Souza, J. A. M. de. Metagenomic Assembly and Draft Genome Sequence of an Uncharacterized Prevotella sp. from Nelore Rumen. *Genome Announc.* **3**, (2015).
10. Taxis, T. M. *et al.* The players may change but the game remains: network analyses of ruminal microbiomes suggest taxonomic differences mask functional similarity. *Nucleic Acids Research* **43**, 9600–9612 (2015).

11. Campanaro, S. *et al.* In vitro fermentation of key dietary compounds with rumen fluid: A genome-centric perspective. *Science of The Total Environment* **584–585**, 683–691 (2017).
12. Li, J. *et al.* A catalog of microbial genes from the bovine rumen unveils a specialized and diverse biomass-degrading environment. *GigaScience* **9**, (2020).
13. Solden, L. M. *et al.* New roles in hemicellulosic sugar fermentation for the uncultivated Bacteroidetes family BS11. *The ISME Journal* **11**, 691–703 (2017).
14. Svartström, O. *et al.* Ninety-nine de novo assembled genomes from the moose (Alces alces) rumen microbiome provide new insights into microbial plant biomass degradation. *The ISME Journal* **11**, 2538–2551 (2017).
15. Ellison, M. J. *et al.* Diet Alters Both the Structure and Taxonomy of the Ovine Gut Microbial Ecosystem. *DNA Research* **21**, 115–125 (2014).
16. Li, R. W. *et al.* Metagenomic Insights into the RDX-Degrading Potential of the Ovine Rumen Microbiome. *PLOS ONE* **9**, e110505 (2014).
17. Shi, W. *et al.* Methane yield phenotypes linked to differential gene expression in the sheep rumen microbiome. *Genome Res.* **24**, 1517–1525 (2014).
