# Supplementary material for: Insights into rumen microbial biosynthetic gene cluster diversity through genome-resolved metagenomics
Source: Commun Biol. 2021 Jun 29;4:818. doi: 10.1038/s42003-021-02331-7 (PMC8241843; doi:10.1038/s42003-021-02331-7)
Supplement: Supplementary file 3 — Description of Supplementary Files [file 42003_2021_2331_MOESM3_ESM.pdf]

Description of Additional Supplementary Files

File Name: Supplementary Data 1
Description: Taxonomy and genomic properties of recovered rumen MAGs. (xlsx)

File Name: Supplementary Data 2
Description: Results from the clustering of MAGs from the current study, the Hungate1000 Collection, and Stewart et al. studies based on 95% ANI thresholds with dRep. (xlsx)

File Name: Supplementary Data 3
Description: BGC predictions for MAGs presented in the current study, genomes from the Hungate1000 Collection, and MAGs from the Stewart et al. studies. (xlsx)

File Name: Supplementary Data 4
Description: Nodes and edges of the relational network based on BiG-SCAPE defined similarity between NRPS gene clusters of Bacteroidota, Firmicutes, and Euryarchaeota. (xlsx)

File Name: Supplementary Data 5
Description: Genome-specific normalized counts and DESeq2 results for 648 BGCs with at least 100 reads and encoded by genomes with at least one read in all 20 metatranscriptomes. (xlsx)

File Name: Supplementary Data 6
Description: Phylogenetic tree of 1,163 near-complete bacterial MAGs recovered in the current study. (Newick)

File Name: Supplementary Data 7
Description: Phylogenetic tree of 20 near-complete archaeal MAGs recovered in the current study. (Newick)

File Name: Supplementary Data 8
Description: Phylogenetic tree of 1,781 near-complete bacterial genomes that were representative genomes of 95% ANI clusters formed from 8,160 rumen-specific microbial genomes. (Newick)

File Name: Supplementary Data 9
Description: Phylogenetic tree of 35 near-complete archaeal genomes that were representative genomes of 95% ANI clusters formed from 8,160 rumen-specific microbial genomes. (Newick)

File Name: Supplementary Data 10
Description: Phylogenetic tree of 1,766 near-complete Firmicutes genomes identified from the 8,160 rumen-specific microbial genomes. (Newick)

File Name: Supplementary Data 11
Description: Phylogenetic tree of 85 near-complete archaeal genomes identified from the 8,160 rumen-specific microbial genomes. (Newick)

File Name: Supplementary Data 12
Description: Phylogenetic tree of 120 near-complete rumen bacterial genomes encoding lanthipeptide BGCs. (Newick)
