# Supplementary material for: Insights into rumen microbial biosynthetic gene cluster diversity through genome-resolved metagenomics
Source: Commun Biol. 2021 Jun 29;4:818. doi: 10.1038/s42003-021-02331-7 (PMC8241843; doi:10.1038/s42003-021-02331-7)
Supplement: Supplementary file 16 — Reporting Summary [file 42003_2021_2331_MOESM16_ESM.pdf]

nature research

Corresponding author(s): Samodha C. Fernando

Last updated by author(s): May 11, 2021

Reporting Summary

Nature Research wishes to improve the reproducibility of the work that we publish. This form provides structure for consistency and transparency in reporting. For further information on Nature Research policies, see our Editorial Policies and the Editorial Policy Checklist.

Statistics

For all statistical analyses, confirm that the following items are present in the figure legend, table legend, main text, or Methods section.

n/a	Confirmed	
☐	☒	The exact sample size (*n*) for each experimental group/condition, given as a discrete number and unit of measurement
☐	☒	A statement on whether measurements were taken from distinct samples or whether the same sample was measured repeatedly
☐	☒	The statistical test(s) used AND whether they are one- or two-sided<br>*Only common tests should be described solely by name; describe more complex techniques in the Methods section.*
☐	☒	A description of all covariates tested
☐	☒	A description of any assumptions or corrections, such as tests of normality and adjustment for multiple comparisons
☐	☒	A full description of the statistical parameters including central tendency (e.g. means) or other basic estimates (e.g. regression coefficient) AND variation (e.g. standard deviation) or associated estimates of uncertainty (e.g. confidence intervals)
☐	☒	For null hypothesis testing, the test statistic (e.g. F, t, r) with confidence intervals, effect sizes, degrees of freedom and P value noted<br>*Give P values as exact values whenever suitable.*
☒	☐	For Bayesian analysis, information on the choice of priors and Markov chain Monte Carlo settings
☒	☐	For hierarchical and complex designs, identification of the appropriate level for tests and full reporting of outcomes
☐	☒	Estimates of effect sizes (e.g. Cohen's *d*, Pearson's *r*), indicating how they were calculated

Our web collection on statistics for biologists contains articles on many of the points above.

Software and code

Policy information about availability of computer code

Data collection	The SRA Toolkit (version 2.8.2) was used to download metagenomic data associated with this manuscript.
Data analysis	BBTools software suite (version 38.16)<br>VSEARCH (version 2.0.3)<br>MEGAHIT (version 1.1.1)<br>SPAdes (version 3.13.0)<br>CheckM (version 1.0.11)<br>Genome Taxonomy Database Toolkit (GTDB-Tk 0.2.2)<br>Genome Taxonomy Database (release 86 v3)<br>Prokka (version 1.13.7)<br>Anvi'o (version 5.5)<br>FastANI (version 1.1)<br>QIIME (version 1.9)<br>Kraken2 (version 2.0.7)<br>antiSMASH (version 4.0)<br>BiG-SCAPE version 20190604)<br>DESeq2 (version 1.24.0)<br>Kallisto (version 0.45.0)<br>InStrain (version 1.2.4)

For manuscripts utilizing custom algorithms or software that are central to the research but not yet described in published literature, software must be made available to editors and reviewers. We strongly encourage code deposition in a community repository (e.g. GitHub). See the Nature Research guidelines for submitting code & software for further information.

Data

Policy information about availability of data

All manuscripts must include a data availability statement. This statement should provide the following information, where applicable:

- Accession codes, unique identifiers, or web links for publicly available datasets
- A list of figures that have associated raw data
- A description of any restrictions on data availability

The accessions for all metagenomes analyzed are available in Supplementary Table 1. Metagenomes previously not publicly available were deposited under NCBI BioProject PRJNA627299 and PRJNA627251. The 2,809 reconstructed MAGs are available at: https://doi.org/10.6084/m9.figshare.12164250.

Field-specific reporting

Please select the one below that is the best fit for your research. If you are not sure, read the appropriate sections before making your selection.

☒ Life sciences ☐ Behavioural & social sciences ☐ Ecological, evolutionary & environmental sciences

For a reference copy of the document with all sections, see nature.com/documents/nr-reporting-summary-flat.pdf

Life sciences study design

All studies must disclose on these points even when the disclosure is negative.

Sample size	As this project was largely discovery-based, sample size was not critical to the outcomes. The samples size of 435 metagenomes is sufficient for metagenomic binning efforts. Other analyses in which sample size was an important factor involved previously published datasets and the process behind sample size selection is described in those cited manuscripts.
Data exclusions	No data were excluded.
Replication	The results of this manuscript are largely discovery-based and replication was not critical to the aims of the project.
Randomization	The first two unpublished metagenomic datasets were from an 84-day growing study utilizing 120 steers and subsequent 125-day finishing study with 60 steers at the University of Nebraska Agriculture Research and Development Center, as described previously (Pesta 2015). From the original 120 animals in the growing study, 23 animals across different treatment groups were randomly selected for metagenomic sequencing. Sixty of the steers were utilized in a finishing study to evaluate the influence of dietary nitrate and sulfate on methane emissions and animal performance. From this study, 27 animals across different treatment groups were selected randomly for metagenomic sequencing.<br><br>Paz et al. (2018) characterized the rumen microbiomes of 125 heifers and 122 steers to identify bacterial operational taxonomic units linked to feed efficiency. From this cohort, 16 steers displaying divergent feed efficiency phenotypes were selected for metagenomic sequencing.<br><br>Other analyses in which randomization was critical involved previously published datasets and the randomization processes are described in those cited manuscripts.
Blinding	Blinding was not relevant to this manuscript as it largely involves previously published datasets.

Reporting for specific materials, systems and methods

We require information from authors about some types of materials, experimental systems and methods used in many studies. Here, indicate whether each material, system or method listed is relevant to your study. If you are not sure if a list item applies to your research, read the appropriate section before selecting a response.

Materials & experimental systems

n/a	Involved in the study	
☒	☐	Antibodies
☒	☐	Eukaryotic cell lines
☒	☐	Palaeontology and archaeology
☐	☒	Animals and other organisms
☒	☐	Human research participants
☒	☐	Clinical data
☒	☐	Dual use research of concern

Methods

n/a	Involved in the study	
☒	☐	ChIP-seq
☒	☐	Flow cytometry
☒	☐	MRI-based neuroimaging

Animals and other organisms

Policy information about studies involving animals; ARRIVE guidelines recommended for reporting animal research

Laboratory animals	This study used data collected from Cattle, Moose, Sheep, Bison and Deer
Wild animals	The study did not involve wild animals.
Field-collected samples	The study did not involve samples collected from the field.
Ethics oversight	The University of Nebraska-Lincoln Institutional Animal Care and Use Committee approved animal care and management procedures.

Note that full information on the approval of the study protocol must also be provided in the manuscript.
